# Supplementary material for: Reasons for reductions in routine childhood immunisation uptake during the COVID-19 pandemic in low- and middle-income countries: A systematic review
Source: PLOS Glob Public Health. 2023 Jan 24;3(1):e0001415. doi: 10.1371/journal.pgph.0001415 (PMC10021156; doi:10.1371/journal.pgph.0001415)
Supplement: S2 Text — (DOCX) [file pgph.0001415.s003.docx]

**S2 Text.** **Changes to Protocol**

This systematic review was planned alongside another review covering the magnitude of disruption to routine vaccinations in low- and middle-income countries, which has since been published. Whilst initially it was planned to report findings together, a substantially greater number results were found than expected, and the authors decided to report reasons for disruption separately to enable greater detail.

The method of synthesis for reasons for disruption initially was planned to be narrative; however, given that over half of studies were cross-sectional (and not qualitative), a quantitative approach was deemed appropriate.

Edits to the databases – that is, the addition of CINAHL and removal of Web of Science – were decided upon consultation with an expert librarian, prior to performing the searches.
