# Supplementary material for: Reasons for reductions in routine childhood immunisation uptake during the COVID-19 pandemic in low- and middle-income countries: A systematic review
Source: PLOS Glob Public Health. 2023 Jan 24;3(1):e0001415. doi: 10.1371/journal.pgph.0001415 (PMC10021156; doi:10.1371/journal.pgph.0001415)
Supplement: S1 Table — (DOCX) [file pgph.0001415.s004.docx]

**S1 Table. Definition of each coded reason for disruptions to routine immunisation**

| **Reason (as coded)** | **Definition** |  |
| --- | --- | --- |
| Fear of COVID-19 | Fear of contracting and transmitting COVID-19, or consequential self-isolation. |  |
| Transport challenges | Issues with transportation to immunisation visits. |  |
| Awareness issues | Service users not aware that immunisation services are available or perception that vaccination is unnecessary (e.g., unaware that an additional dose of vaccine was needed). |  |
| Lockdown policy | Policies or guidelines encouraging individuals to stay home and avoid public spaces Not including self-isolation or illness. |  |
| Personal or non-pandemic related | Any issue that prevents individual from attending immunisation that is due to a personal circumstance (e.g. going on holiday). |  |
| Inadequate staffing | Refers to an under-provision of staff. May be a consequence of COVID-19 pandemic, or for unspecified reasons. |  |
| Inactive immunisation service | Where whole vaccine programs or services were shut down or had reduced activity. |  |
| Sparing healthcare services | Personal choice to avoid healthcare unless strictly necessary to help spare services during the pandemic. |  |
| Financial challenges | Financial difficulties faced during pandemic. |  |
| Scheduling challenges | Issues with scheduling of appointments that prevented immunisation visit. |  |
| Discouragement from others | Pressure from others, including family members and neighbours, not to attend immunisation services. |  |
| Social challenges | Refers to personal and socially-based consequences of pandemic (e.g. increased stress, general fear of public spaces, family issues). |  |
| Inadequate vaccine supply | Refers to an unavailability of vaccine supplies. |  |
| Self-isolation or illness | Parent or child ill and therefore unable to attend vaccination. |  |
| Fear of side-effects | Fear of side-effects and medical consequences of vaccine. |  |
| Inadequate Personal-protective equipment (PPE)* | Refers to an unavailability of PPE affecting immunisation activity. |  |
| Lack of guidance | Issues in guidelines or leadership regarding continuation of vaccination services during the COVID-19 pandemic. |  |

**PPE: clothing, accessories or other garments worn with aim of protecting from infection. For example: masks, goggles, gloves.*
