# Supplementary material for: Reasons for reductions in routine childhood immunisation uptake during the COVID-19 pandemic in low- and middle-income countries: A systematic review
Source: PLOS Glob Public Health. 2023 Jan 24;3(1):e0001415. doi: 10.1371/journal.pgph.0001415 (PMC10021156; doi:10.1371/journal.pgph.0001415)
Supplement: Attachment — Submitted filename: Response Letter.docx [file pgph.0001415.s006.docx]

Dear Editorial Team,

Thank you for your responses and for the opportunity to revise our manuscript.

We have carefully considered all points raised by reviewers and revised the manuscript accordingly. Our amendments can be found as tracked changes on the resubmitted manuscript and are also detailed below, in blue. Page and line numbers refer to the re-uploaded manuscript with tracked changes.

Yours faithfully,

Alexandra Pinto (on behalf of all co-authors)

**Editor comment 1**
1. Please review your reference list to ensure that it is complete and correct. If you have cited papers that have been retracted, please include the rationale for doing so in the manuscript text, or remove these references and replace them with relevant current references. Any changes to the reference list should be mentioned in the rebuttal letter that accompanies your revised manuscript. If you need to cite a retracted article, indicate the article’s retracted status in the References list and also include a citation and full reference for the retraction notice.

**Editor response 1**

*All references have been reviewed and formatting edited as applicable. All formatting changes have been tracked.*

**Editor comment 2**

2. Please insert an Ethics Statement at the beginning of your Methods section, under a subheading 'Ethics Statement'. It must include:

1) The name(s) of the Institutional Review Board(s) or Ethics Committee(s)

2) The approval number(s), or a statement that approval was granted by the named board(s)

3) (for human participants/donors) - A statement that formal consent was obtained (must state whether verbal/written) OR the reason consent was not obtained (e.g. anonymity). NOTE: If child participants, the statement must declare that formal consent was obtained from the parent/guardian.

**Editor response 2**

*Systematic reviews do not require ethical approval. This has been stated at the beginning of the Methods section, under the heading “Ethics Statement”:*

*“As this work is a systematic review, ethics approval was not required.” (p. 5 lines 136-137)*

**Editor comment 3**

3. We have noticed that you have uploaded Supporting Information files, but you have not included a list of legends. Please add a full list of legends for your Supporting Information files after the references list.

**Editor response 3**

*A full list of legends for supporting information files has been added after the references list.*

**Editor comment 4**

4. In the online submission form, you indicated that "All data has been shared as supplementary files. Further clarifications can be made available upon request to corresponding author". All PLOS journals now require all data underlying the findings described in their manuscript to be freely available to other researchers, either 1. In a public repository, 2. Within the manuscript itself, or 3. Uploaded as supplementary information.
This policy applies to all data except where public deposition would breach compliance with the protocol approved by your research ethics board. If your data cannot be made publicly available for ethical or legal reasons (e.g., public availability would compromise patient privacy), please explain your reasons by return email and your exemption request will be escalated to the editor for approval. Your exemption request will be handled independently and will not hold up the peer review process, but will need to be resolved should your manuscript be accepted for publication. One of the Editorial team will then be in touch if there are any issues.

**Editor response 4**

*All data has been uploaded as Supplementary File 4: Data.*

Reviewer #1: Overall, this is a timely review on the impact of COVID-19 on routine immunisation in low and middle income countries globally. Specific comments are provided by each section of the paper below.

**Reviewer 1 comment 1**
Abstract
- Line 41: Some evidence of recovery/bounce back in coverage, including in LMICs, has been noted recently e.g. <https://www.gavi.org/news/media-room/immunisation-lower-income-countries-pandemic-leads-decline-coverage-signs-recovery>

**Reviewer 1 response 1**

Thank you for raising this point. The statement “with no evidence of recovery” is now outdated and has therefore been removed. (p. 2, line 41)

**Reviewer 1 comment 2**
- Line 50: NHLBI and CASP tools recommended to be spelt out in full at first use

**Reviewer 1 response 2**

These have now been spelled out. (p. 2 lines 50-51)

**Reviewer 1 comment 3**
- Line 57: Unclear how the (n=4246) is relevant - not until later in the text that this is reported to be the total number of participants. Suggest removing here or including "with a total of XX participants" after "Thirteen studies..."

**Reviewer 1 response 3**

Thank you for this point. The abstract has now been edited to reflect the quantitative and qualitative results separately; participant numbers for each are also provided. For example, “In quantitative studies (total respondents = 2,853)” (p. 2 line 59; p 2-3 lines 64-82)

**Reviewer 1 comment 4**
- Line 66: 'Reason for disruption' could be made clearer e.g. most common contributors to reduced routine immunisation coverage

**Reviewer 1 response 4**

Thank you for this point. We hope to have made this clearer by providing a succinct definition in the methodology section, which reads (p6, lines 152-153):

*“Primary research studies exploring reasons for disruptions to immunisation programmes were included, where ‘reasons for disruptions’ is defined as any factor that may have contributed to declines in routine immunisations, as reported by studies (Figure 1)”*

Where italics indicate sections extracted from the revised manuscript and underlined phrases indicate revisions.

**Reviewer 1 comment 5**

- The term 'LMICs' is very broad (and therefore somewhat meaningless), and the included studies hardly consider Central/South America, and do not include any data from the Pacific. While you note this limits generalisability in your discussion, it is important to prevent over-reaching in your framing throughout the article. These articles may also be insightful: <https://gh.bmj.com/content/7/6/e009067>; <https://gh.bmj.com/content/7/6/e009704>

**Reviewer 1 response 5**

Thank you very much for highlighting this issue. Whilst taking a broad approach enables a wide range of identified issues, it may also limit the usefulness, or ability to provide specific, tailored conclusions. Furthermore, given the limited number of represented world regions (which are also themselves already broad), and countries, it could be argued that despite taking a broad approach, results continue to be limited in scope.

The choice of focusing on LMICs stemmed from previous work by the same group. It also provided a specific economic definition which allowed the inclusion/exclusion of articles in a straightforward way. That is not to say that this could not have been achieved using other approaches but, at the time of designing the protocol, this was the decision made.

It is a significant limitation that we agree needs to be discussed and acknowledged further. Whilst the paper previously mentioned issues with generalisability, we hope to further explain and highlight this issue in the discussion, as follows:

*“Additionally, the decision to focus on LMICs is in itself very broad, which may challenge the ability to establish specific, effective locally meaningful conclusions; in future, more targeted choices of location or subgroup analyses would be beneficial.”* (p.17-18 lines 467-472) where the first article recommended by Reviewer 1 is referenced.

The section continues to explain additional reasons that may limit generalisability of results:

*“There may be local factors that affect disruptions to vaccination, such as differing approaches to COVID-19 mitigation strategies, previous levels of vaccination coverage, accessibility of healthcare and even cultural factors. Therefore, whilst this review may be helpful in providing an overview of key issues, strategies to overcome identified issues must be tailored to local contexts.”* (p. 18 lines 472-476).

Finally, we hope to emphasise that whilst this review can provide a helpful overview of broad issues and synthesise evidence from previous global health emergencies, ultimately the solutions need to be targeted to local populations – a decision that can only be made and best implemented by those in the community themselves:

*“It is also vital to note that for strategies to be effective and targeted, they should be chosen, designed, and implemented by local teams or authorities.”* (p. 18 lines 476-478).

**Reviewer 1 comment 6**

Background
- Line 71: Difficult to assess that children have been spared 'consequences of severe COVID-19'; for instance, what about sick or dying family members, disruptions to schooling etc? While your focus may be on clinical manifestations of COVID-19, this phrasing seems dismissive and I suggest rewording

**Reviewer 1 response 6**

Thank you for this point. Indeed, children have *not* in any way been spared of indirect consequences of the pandemic, e.g. sick or dying family members, disruptions to schooling, disruptions to healthcare, amongst many others. The phrase referred to clinical manifestations of COVID-19 on themselves, and as such has been rephrased to:

*“Although children have largely been spared the consequences of severe clinical manifestations of COVID-19, they have been subject to several significant indirect effects of the pandemic”* (p. 4, lines 91-93).

**Reviewer 1 comment 7**
- Reference 8 does not address declines in routine immunisation due to COVID-19, and reference 9 is too preliminary to provide good evidence to support this. While I believe that COVID-19 impacted on immunisation services, stronger supporting references are needed

**Reviewer 1 response 7**

Thank you for highlighting these issues. The purpose of this sentence was to emphasise that drops in routine vaccinations can lead to setbacks in elimination of vaccine-preventable diseases and increase the risk of outbreaks. As such, the sentence has been amended to state *“Such declines have resulted in significant setbacks in elimination of vaccine-preventable diseases such as measles and polio (8,9)”* (p. 4 lines 95-96), with references (8) and (9) being WHO press releases highlighting outbreak of polio in Malawi and general setbacks in measles elimination during the COVID-19 pandemic, respectively.

**Reviewer 1 comment 8**
- Line 86: Your suggestion that families 'chose' not to vaccinate their children fails to take into account the myriad barriers to accessing routine immunisations and is not supported by global evidence - zero-dose children are overwhelmingly due to access barriers, not hesitancy

**Reviewer 1 response 8**

Thank you very much for this point. The purpose of the sentence was to introduce the idea of vaccine hesitancy – however, placing it before the number of zero-dose children does indeed suggest that the major cause of no vaccination is due to hesitancy, which is inaccurate. As such, this has now been rephrased:

*“Nevertheless, even prior to the COVID-19 pandemic, many children remained unvaccinated; in 2019, UNICEF estimates that 13 million children received no dose of the DTP vaccine – this value increased by 39% in 2021” (p. 4 lines 104-106).*

**Reviewer 1 comment 9**

Methods
- The link to the previously published paper by the same group (ref 7) should be made more explicit (this could also happen where it is discussed in the background section). At times, the article felt a little lacking substance and splitting this topic in two would help explain - but not justify - why that is the case.

**Reviewer 1 response 9**

*Thank you for this comment. The final paragraph of the introduction already discusses the findings of the previous systematic review, followed by the aims of the current study. We have added a sentence to make explicit that one follows from the other, which we hope makes the link between the two clearer. This now reads:*

*“A previous systematic review summarised the extent of disruption (…) and found a decline of over 10% in all vaccines universally recommended by the World Health Organisation (…). Therefore, and following on from the findings of the previous systematic review, the aim of this study was to summarise the reasons behind disruptions (…)” (p. 5 lines 127-128).*

**Reviewer 1 comment 10**

- I am unclear about data availability - there is no attached/supplementary file which includes the calculation of the means for instance. despite stating in the data availability statement that all data have been shared as supplementary files. The completed "pre-defined Excel spreadsheet' (line 142) has also not been shared

**Reviewer 1 response 10**

*Thank you for highlighting this – the supplementary file has now been uploaded, containing extraction and analysis sheets (Supplementary File 4: Data).*

**Reviewer 1 comment 11**
- Line 149: is there any justification for why NHLBI and CASP were chosen to assess study quality?

**Reviewer 1 response 11**

*These tools were chosen because, when the original protocol was developed, they were thought to be comprehensive enough to cover a broad range of possible study designs. This is now explained in the manuscript:*

*“These tools were chosen as they were found sufficiently comprehensive to cover a range of possible study designs at the time the protocol was designed.”* (p. 7, lines 180-181).

**Reviewer 1 comment 12**

- Line 167: Why do you try to quantify qualitative data? It is a big assumption that, even with a small sample size, every participant reported every issue and is not recommended

**Reviewer 1 response 12**

Thank you for this point, which highlights a very valid concern. This assumption applied to three findings: proportion of responses by healthcare-seeking and healthcare-delivery issues; by location; by stakeholder.

The findings by location and stakeholder have been adapted so that they no longer apply to the proportion of responses, but rather to the proportion of respondents. This was possible to do as studies provided respondents by location and detailed who the respondents were.

However, the same could not always be done for estimating the proportion of responses that apply to healthcare-seeking and healthcare-delivery issues, as some studies did not report how many participants stated each reason. Although this method relies on a big assumption, it enables us to compare the proportion of healthcare-seeking and healthcare-delivery issues by taking into account both how many reasons were reported for each and also the sample size of the study.

**Reviewer 1 comment 13**

Results
- Table 2: The number of studies and the number of references on several lines do not match up (e.g. time period; also extends beyond 2020 in Bimpong et al). Would be good to highlight that the categories are generally not mutually exclusive

**Reviewer 1 response 13**

Thank you for highlighting this – the table has now been corrected. We have also added a footnote to the table to explain that *“Characteristics are not mutually exclusive.”* (p. 9 line 228).

**Reviewer 1 comment 14**
- Figure 2: Caption does not adequately explain process. What does +/-/? mean? How were the scores determined? Graphic also unclear/poor quality image

**Reviewer 1 response 14**

This caption has now been amended to more adequately explain the meaning of the symbols used:

*“Results of quality assessment for included studies, following NHLBI and CASP assessments criteria, where (+) denotes that a criterion was met, (-) denotes that it was not met, and N/A denotes the criterion was not applicable to the study.”* (p. 21, Figure 2 legend).

More detail has also been added to the relevant section of the methods to explain how the appraisal took place:

*“Reviewers assessed the quality of studies against the relevant checklist, noting if each criterion was met, not meet, or not applicable, followed by an overall quality score (poor, fair, good).”* (p. 7, lines 183-185).

The graphic has also been re-uploaded and is of higher quality than the original.

**Reviewer 1 comment 15**
- Line 202: 'failure to immunise' - similar to line 86, the loaded language has strong implications and more considerate terminology regarding the barriers faced should be used

**Reviewer 1 response 15**

Many thanks for highlighting this – the phrasing has now been adapted to *“most reported reason for missing immunisations was (…)” (p. 12 line 285).*

**Reviewer 1 comment 16**
- Fig 4: NA category not clear. Order should match Supp Table A1 (personal barrier category moves position - there should also be some discussion of this later in the paper)

**Reviewer 1 response 15**

The NA category applied to causes that were neither healthcare seeking or healthcare delivery issues. For instance, parents forgetting about appointments for vaccination, or going on holiday. These were all under the category of “personal or non-pandemic related”, as per figure 5. This has been briefly explained in the caption for Figure 5*: “A minority of causes did not apply to either category and were denoted as N/A.”* (p. 21, Figure 6 legend).

Figure 4 was organised by order of decreasing frequency. Supplementary Table A1 (Supplementary File 3). has been adjusted to match the order of the figure.

**Reviewer 1 comment 17**
- Line 216: 34 coded causes - does this mean 34 different reasons were identified? How does this fit into the 17 reported for the cross-sectional papers?

**Reviewer 1 response 17**

Thank you for highlighting this. The 34 different reasons refer to the extracted data. This has now been reworded in the manuscript to state *“extracted reasons”* (p. 13, lines 322-323). Please note there was also an error in the original submission, as the number of extracted reasons was 35, not 34.

After all data (or reasons) were extracted from studies, these were analysed by reviewers and grouped into categories, enabling us to match similar reasons. By the end of this analysis, 11 final coded reasons were identified in the qualitative papers.

A similar process is now described for the quantitative studies, which identified 17 unique reasons:

*“A total of 74 reasons were extracted and from these 17 unique reasons for disruptions to routine immunisation were coded”* (p. 12 lines 281-282).

**Reviewer 1 comment 18**
- Line 222-3: Unclear if a 'lac of guidance regarding delivery of routine vaccines' is referring to routine immunisation handbooks/guidelines, or Covid-specific information regarding PPE, service provision etc

**Reviewer 1 response 18**

This has now been clarified and states: *“(…) and lack of guidance regarding the delivery of routine vaccines by HCPs in the context of the COVID-19 pandemic including safety precautions and clear plans for service-provision”* (p. 13 lines 332-333).

**Reviewer 1 comment 19**
- Good addition of facilitators to routine immunisation in one study (lines 231-8). Did any other papers include this? Would be interesting to further discussion

**Reviewer 1 response 19**

Thank you for this point. Despite this not being the focus of the study, one other study did note factors that were found not to impact routine vaccination (Bimpong et al. 2021). This has now been added to this section, and corroborate findings from the previously described study.

*“Another study also found that unavailability of vaccines or poor staffing were not reasons for missed routine immunisations as despite the reallocation of staff towards COVID-19 efforts, routine vaccination services remained staffed and parents did not note any shortages of vaccine supplies.”* (p. 14 lines 355-358).

**Reviewer 1 comment 20**
Discussion
- Further discussion about the breakdown of specific barriers by respondent status (family or HCP) would be useful - you state in line 166 that barriers are subdivided by reporting stakeholder but this is not clear. For instance, how health-seeking vs health-delivery challenges were determined could do with some more fleshing out - there is some confusion around transport-related barriers

**Reviewer 1 response 20**

Thank you for this point. Figures 3 and 4 now depict the proportion of respond*ents* that were parents or HCPs. It is important to highlight this as it may affect the proportion of answers that are healthcare-seeking or healthcare-delivery issues.

The terms healthcare-delivery and healthcare-seeking are defined in p. 8, lines 194-197; they are similar terms to ‘supply’ and ‘demand’ – as used by the World Health Organization. Our findings are also compared with WHO pulse survey findings in the discussion (p. 15 lines 369-377).

The classification of healthcare-seeking and healthcare-delivery – and in particular how a single coded cause could at times apply to both – is now described in more detail in lines X-Y, which read as follows:

*“While many coded causes were entirely classified as either health-seeking or health-delivery issues, some were classified with both. For instance, transport challenges which affected HCPs ability to attend work was classified health-delivery issue, whereas transport challenges that described patients being unable to attend services were classified as health-seeking issues. Similarly, fear of infection could apply to both HCPs and patients – although the vast majority, X%, applied to parents – as could financial challenges, and scheduling challenges.”* (p. 12 lines 295-318).

**Reviewer 1 comment 21**

-Line 276-7: how does living far from immunisation services drive vaccine hesitancy? This paragraph would be better later in the discussion. as hesitancy did not appear to be a significant factor identified in your review. Similarly around line 314 the focus on counteracting hesitancy (rather than generally removing barriers to access) seems misplaced

**Reviewer 1 response 21**

This paragraph has now been shifted to the end of the discussion (p. 18 lines 490 onwards). Additionally, it has been further developed to emphasise that whilst vaccine hesitancy is an important consideration, barriers to accessing vaccinations – which also existed pre-pandemic – are also vital to consider. Distance from health services is better described as an accessibility issue than hesitancy.

*“Additionally, although the findings of this review focus on pandemic-related drivers of reduced vaccination, vaccine hesitancy was already a concern prior to the COVID-19 pandemic and hence previously identified, non-pandemic-related factors driving vaccine hesitancy are likely to remain relevant. These may include disbelief in vaccines or severity of vaccine-preventable disease, distrust of health professionals or fear of vaccine side-effects. Discussions for and against the use of COVID-19 vaccines – especially between health professionals and politicians – may have also heightened hesitancy towards other vaccines. One survey-based study found increased hesitancy and heightened perception of routine immunisation risks amongst parents at the Children’s Hospital Los Angeles during the pandemic. Furthermore, previously identified barriers to vaccination are likely to also remain relevant, such as living far from immunisation services and associated long duration of travel and associated costs, language and cultural barriers, lack of reminders and poor support from healthcare institutions, amongst several others. This highlights the importance of considering additional factors underlying both vaccine hesitancy and barriers to accessing healthcare when designing efforts for immunisation recovery, and of monitoring the impact of the pandemic on routine paediatric vaccine confidence.” (p. 18-19, lines 490-509).*

**Reviewer 1 comment 22**

- Line 298-302: Feasibility of all these strategies - providing PPE, home visits, and school programs (especially when schools were shut in many countries for a long period) must be considered in the context of the countries you are discussing - these solutions are often costly and may not be feasible in financially-constrained settings

**Reviewer 1 response 22**

Thank you for this point. It is true that different countries – and even regions within a single country – are likely to have different available resources, and priorities. We hope to have emphasised this and the importance of tailored interventions in the discussion, as follows:

*“In practice, the most effective and feasible methods to reach communities and deliver vaccinations safely will vary between and within countries. Approaches should be adapted to their target population based on local needs, available resources and infection control guidelines.*” (p. 17, lines 454-457).

This point is also highlighted after discussing limitations on representation in this study (p. 18, lines 472-476):

*“There may be local factors that affect disruptions to vaccination, such as differing approaches to COVID-19 mitigation strategies, previous levels of vaccination coverage, accessibility of healthcare and even cultural factors. Therefore, whilst this review may be helpful in providing an overview of key issues, strategies to overcome identified issues must be tailored to local contexts.”*

**Reviewer 1 comment 23**

- Some additional discussion or examples of the types of 'strong public health messaging' you suggest in your concluding paragraph (line 333) would strengthen your discussion

**Reviewer 1 response 23**

Thank you for this point. The second half of the discussion focuses on recommendations for recovery of missed immunisations, often based on strategies described in previous outbreaks, whilst also emphasising that any chosen strategy must be tailored to target populations. This is especially important considering the breadth of this review, namely the inclusion of “low *and* middle-income countries”, which as we have acknowledged earlier, is extremely broad. We hope, however, to have made the point clearer by reviewing it to say as follows:

*“As nations develop efforts to recover missed immunisations, it is essential to invest in measures that ensure continuity of safe vaccine delivery alongside strong public health campaigns advertising availability and safety of routine vaccination to encourage routine immunisation uptake and attendance to health-services, even in the context of a pandemic.” (p.19 lines 514-517).*

Reviewer #2: This is a thoroughly researched and well written article.
The topic matter is timely and of utmost importance in relation to child health outcomes globally.
Findings should be disseminated widely to promote global efforts to increase routine vaccination in the most vulnerable populations.

**Reviewer 2 comment 1**
Please consider some minor points to review:
Background - sets scene nicely to emphasis drop in immunisation rates, especially in LMICs. Paragraph 2 - consider rephrasing "families chose not to vaccinate" as this implies vaccine hesistancy/anti-vaxxer sentiments and precludes other reasons such as access issues (that you later go onto discuss).

**Reviewer 2 response 1**

Thank you for this point. This phrase is now reviewed to say: *“Nevertheless, even prior to the COVID-19 pandemic, many children remained unvaccinated”* (p. 4, lines 104-105).

**Reviewer 2 comment 2**
Results
- Overall, there is large difference of proportion between the healthcare seeking challenges reasons (81.9%) and the healthcare delivery challenges (15.8%). It might make more impact on this difference if you present results this way. This is demonstrated /visualised well in figure 5 but not discussed that way in your written results section.

**Reviewer 2 response 2**

Thank you for this point. Figures 3 and 4 now contain a pie chart highlighting these proportions, hopefully making them clearer. They are also discussed at the start of the results section for both quantitative and qualitative studies.

**Reviewer 2 comment 3**
- your qualitative studies show relative equal proportion between health seeking and health delivery reasons. But I can't see what the proportions are in the quantitative studies? I assume more weighted towards health seeking challenges to have overall results tip that way.

**Reviewer 2 response 3**

Thank you for this point. Given that most of the results were already divided into quantitative and qualitative findings, separately, Figure 3 has been edited to separate data for quantitative and qualitative findings as well. Instead, there are now two figures, one for each section.

Additionally, we have added a similar pie chart depicting the proportion of responses that were suggestive of healthcare-seeking or healthcare-delivery issues.

**Reviewer 2 comment 4**
Discussion
- Has the difference in ways data is collected between quantitative and qualitative studies affected the results? If findings are quite disparate, which type of studies should we rely more on for policy/program development?

**Reviewer 2 response 4**

Thank you for this interesting point. In reality, probably a combination – quantitative studies are likely to provide more representative views of issues (as they usually have larger sample sizes), however qualitative studies provide helpful context and in-depth review of identified issues.

This is now also highlighted in the discussion:

*“This review also summarised both quantitative and qualitative findings. Whilst there was substantial overlap in the reasons identified in these two subgroups, quantitative studies had greater proportion of healthcare-seeking issues than qualitative studies. This may be due to the study participants, as there were more HCP respondents in qualitative studies than quantitative, but also likely due to the limitations of assuming that all participants reported each reason (unless otherwise specified) in qualitative studies. When using evidence for policy change, it is vital to include both types of evidence together; quantitative findings may provide a more representative overview of issues, but qualitative findings help provide context and explain these issues more in depth.”* (p. 18, lines 480-488).

**Reviewer 2 comment 5**
- The results included WHO regions and countries where the studies were from. However, there is no analysis of this in the discussion. Are there particular features/ aspects from these countries (eg. cultural beliefs, local policy on COVID mitigation methods), that may have affected the results? As these reasons would help targeted interventions for those countries/regions.

**Reviewer 2 response 5**

Thank you for this point. The decision not to discuss results by region was deliberate; whilst the review aimed to identify key issues leading to vaccine disruptions globally, there was not an even distribution of studies by WHO world region – or even by countries within each world region. Therefore, any such subgroup analyses would be limited in their value. However, we hope to have discussed this limitation, and the implications of having globally-representative reviews, in p.17-18, lines465-478:

*“Moreover, despite the review aiming to summarise reasons globally, a limited number of countries are represented in this study, and even within each country, only limited samples of the relevant population were included, limiting generalisability.* *Additionally, the decision to focus on LMICs is in itself very broad, which may challenge the ability to establish specific, effective locally meaningful conclusions; in future, more targeted choices of location or subgroup analyses would be beneficial. There may be local factors that affect disruptions to vaccination, such as differing approaches to COVID-19 mitigation strategies, previous levels of vaccination coverage, accessibility of healthcare and even cultural factors. Therefore, whilst this review may be helpful in providing an overview of key issues, strategies to overcome identified issues must be tailored to local contexts.* *It is also vital to note that for strategies to be effective and targeted, they should be chosen, designed, and implemented by local teams or authorities.”*

**Reviewer 2 comment 6**

References
Ensure references follow appropriate referencing style (Vancouver). There shouldn't be pdfs attached to end of reference; websites/online articles should have date cited and "available from" prior to URL

**Reviewer 2 response 6**

Thank you for highlighting this – references have been reviewed and generated using EndNote. They should be all corrected.
